# Supplementary material for: Lower-Limb Muscle Power Is Negatively Associated with Protein Intake in Older Adults: A Cross-Sectional Study
Source: Int J Environ Res Public Health. 2022 Nov 7;19(21):14579. doi: 10.3390/ijerph192114579 (PMC9653676; doi:10.3390/ijerph192114579)
Supplement: Supplementary file 1 [file ijerph-19-14579-s001.zip › ijerph-1984100-supplementary.pdf]

Table S1. Participant distribution according to body mass index categories

	Men (34)	Women (163)
Underweight	7 (20.6)	10 (6.1)
Normal-weight	10 (29.4)	42 (25.8)
Overweight	17 (50.0)	111 (68.1)

Data are numbers (%)

Table S2. Linear regression for the association between muscle power and protein-related parameters

	Absolute muscle power		
	Adjusted*		
Variables	Unstandardized coefficients	P value	95% CI
Protein intake/body weght	–28.4	0.01	–49.8, –7.0
Lunch protein intake	–10.4	0.44	–37.7, 16.0
	Allometric muscle power		
	Adjusted*		
Variables	Unstandardized coefficients	P value	95% CI
Adjusted protein intake	–0.09	0.02	–0.17, –0.01
Lunch protein intake	–0.03	0.54	–0.14, –0.07

*Adjusted for age, body mass index, sex, and kilocalories; CI = Confidence interval.

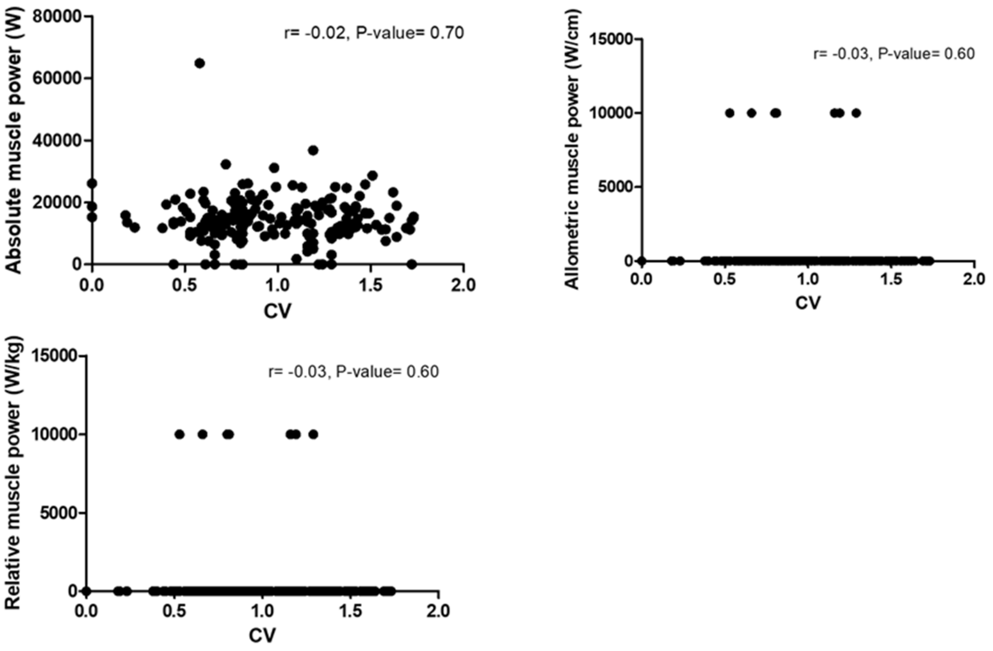

Figure S1. CV, coefficient of variation = standard deviation of g of protein intake per main meal/average total amount of protein (g) of the main meals.
